# Online cognitive testing in Parkinson’s disease: advantages and challenges

**DOI:** 10.3389/fneur.2024.1363513

**Published:** 2024-04-08

**Authors:** Sharon Binoy, Avigail Lithwick Algon, Yoad Ben Adiva, Leila Montaser-Kouhsari, William Saban

**Affiliations:** ^1^Loyola Stritch School of Medicine, Maywood, IL, United States; ^2^Center for Accessible Neuropsychology and Sagol School of Neuroscience, Tel Aviv University, Tel Aviv, Israel; ^3^Department of Occupational Therapy, Faculty of Medical & Health Sciences, Tel Aviv University, Tel Aviv, Israel; ^4^Department of Neurology, Brigham and Women Hospital, Harvard University, Boston, MA, United States

**Keywords:** online, cognitive testing, early detection, Parkinson’s disease, basal ganglia

## Abstract

Parkinson’s disease (PD) is primarily characterized by motor symptoms. Yet, many people with PD experience cognitive decline, which is often unnoticed by clinicians, although it may have a significant impact on quality of life. For over half a century, traditional in-person PD cognitive assessment lacked accessibility, scalability, and specificity due to its inherent limitations. In this review, we propose that novel methods of online cognitive assessment could potentially address these limitations. We first outline the challenges of traditional in-person cognitive testing in PD. We then summarize the existing literature on online cognitive testing in PD. Finally, we explore the advantages, but also the limitations, of three major processes involved in online PD cognitive testing: recruitment and sampling methods, measurement and participation, and disease monitoring and management. Taking the limitations into account, we aim to highlight the potential of online cognitive testing as a more accessible and efficient approach to cognitive testing in PD.

## Introduction

### Importance of cognitive testing in PD

As life expectancy increases, the global population of older adults is growing. This demographic shift leads to a rise in age-related diseases, such as Parkinson’s disease (PD). PD is the most common age-related motor neurodegenerative condition (1). PD is clinically diagnosed primarily by motor impairments (2). However, nonmotor symptoms are increasingly recognized as characteristic of PD, with a decline in cognition being particularly dominant (3). Cognitive decline is often covert or unnoticed by clinicians and patients but has a significant impact on quality of life and daily functioning (3). In fact, about half of the PD population develops dementia within 10 years of diagnosis (4). By understanding the cognitive changes in PD, researchers and clinicians can develop better diagnostic tools and interventions to improve the well-being of people with PD (5). However, traditional in-person cognitive assessment tools have several limitations. To overcome these limitations, there has been a widespread interest in methods to conduct cognitive assessment online.

In this review, we will define and outline major challenges in traditional face-to-face (F2F) PD cognitive testing, overview current cognitive assessments utilizing online methods, and discuss the advantages and limitations of online cognitive testing in people with PD.

### Challenges of current face-to-face cognitive testing methods in PD

Despite the importance of cognitive assessment in PD, most existing F2F testing methods fail to meet global needs. Since the aging population is rapidly growing in many countries, and PD is the most common motor neurodegenerative condition among older populations worldwide, it is essential to expand the accessibility of current cognitive assessments (6, 7). In Table 1, we summarize the advantages and challenges of traditional in-person cognitive assessment in PD.

**Table 1:** Advantages and challenges of traditional in-person cognitive assessment in PD.

Advantages	Challenges
Interpersonal interactions: Feel more genuine, and engagement levels are higher. Privacy: Patients perceive there is more privacy of medical information. Conditions: Controlled test conditions. Adaptability: Alter testing and diagnosis based on verbal and non-verbal cues.	Accessibility: Motor and cognitive symptoms may pose a difficulty. Scalability: Often small sample size (*n* < 30). Diversity: The population is mostly homogenous, not including participants from rural areas or underrepresented populations in PD studies. Completion time: It can take more than 1 year to complete a single PD experiment.

It is often challenging to identify and recruit patients for in-person cognitive assessments, especially those with motor and mobility impairments, such as PD (8). Given their medical condition, patients may also have limited time and energy to participate in studies (9, 10). Motor impairments make participation challenging since cognitive assessments often require the usage of a pen and paper or a non-personalized computer keyboard or mouse. Also, people with PD have specific motor impairments, such as bradykinesia and rigidity, that make traveling distressing or inconvenient for participants. PD affects mobility and balance, limiting the ability to use public transportation, travel long distances, and navigate crowded spaces.

In addition to motor impairments, cognitive and behavioral impairments can make in-person participation challenging. Cognitive deficits in PD include difficulty with executive functions, planning, and attention. Roughly 25% of individuals with PD are diagnosed with mild cognitive impairments, and 24%–31% with dementia (11). Thus, individuals with PD may find it challenging to navigate and commute to labs and clinics. Given the decreased inhibition linked to PD, PD-associated impulsivity (12) may also lead to difficulty in participating, and participants may find the familiar environment of their own home to be less aggravating for their non-motor symptoms.

Also, assessments usually require experts, such as neuropsychologists and neurologists, who are often costly and in high demand (13, 14). This issue is especially pronounced for people who live in remote areas where there are fewer resources and services available. Since PD is a progressive disease, consistent long-term assessment is necessary for clinicians to monitor patient progress, which can be challenging with in-person assessment. In addition, studies conducted on PD patients tend to have small sample sizes, frequently with 30 or fewer participants from the same geographic location, often centered in urban areas with research institutions (15, 16). This leads to biased samples that may not represent the entire PD population. For example, in-person studies tend to disproportionately include PD patients with higher education levels (17, 18).

One potential solution to the challenges of F2F methods is to leverage the Internet. In recent years, behavioral researchers in different domains are increasingly using the Web to reach larger (19) and more diverse (20) populations. Numerous fields of psychology have tapped into the power of crowdsourcing platforms to efficiently obtain valid behavioral data (21). Clinical psychology (22) and developmental studies (23), which have limited in-person access to their research population, have particularly benefitted. Online platforms, however, were not designed for neuropsychological testing/research, and none have been designed specifically for the PD population. To date, online methods have been used in a limited manner in medical (24) and neuropsychological research, with a focus on collecting survey data (25) or for patient recruitment (26).

### Current implementations of online cognitive methods in PD

Next, we review current literature focusing on online cognitive testing of people with PD. We provide examples of the use of online cognitive assessment protocols, videoconferencing (VC), smartphone-based, self-report online surveys, and data collection from online patient-centered forums. In Table 2, we summarize papers reporting online studies assessing cognition in PD.

**Table 2:** Online studies that assessed cognition in PD.

Authors (year)	Method	Domain/task	Population (*n*)	Main advantage	Main disadvantage
Saban and Ivry (27)	Videoconferencing (VC) and fully automated remote testing	MoCA and sequence learning task	SCA (spinocerebellar ataxia) (103)	Efficient data collection in a large and diverse participant pool; May adapt to other patient groups.	No direct comparison to in-person data
			PD (133)		
			Control (159)		
Binoy et al. (28)	Videoconferencing (VC) and fully automated remote testing	MoCA, Anxiety, Depression, Interpersonal Support, personality traits	Experiment 1: PD (73), CA (60), Control (39)	Demonstrates the feasibility and efficiency of online testing to study 5 different domains in 3 groups.	Small sample size in Experiment 2, and no direct comparison to in-person testing
			Experiment 2: PD (16), CA (18), Control (16)		
Stillerova et al. (29)	Face-to-face vs. VC	MoCA	PD (11)	Convenience and efficiency of VC	Participants reported concern for those without access to or skill with a computer.
Constantinescu et al. (30)	VC	Motor and nonmotor: speech	PD and Hypokinetic Dysarthria (61)	Online assessments of speech are valid and reliable for PD.	No control participants
					Not fully automated, requires a rater
Binoy et al. (31)	VC	MoCA	PD (40), CA (40), control (40)	Demonstrated validity of remote MoCA testing in Israel and the USA.	No direct comparison between online and in-person testing
Saban et al. (32)	A fully automated online task	Arithmetic verification task	Experiment 1: PD (18), CD (17), control (20)	Demonstrates the feasibility of administering a fully remote experiment on PD, including recruitment, motor and cognitive assessment, and experimentation	Limited sample size (<20)
			Experiment 2: PD (17), CD (20), control (20)		
Schneider et al. (33)	A virtual longitudinal study (AT-HOME PD)	MoCA, mPower 2.0 Smartphone App, PDQ-8, NMS-Quest, Parkinson’s Disease-Patient report of Problems (PD-PROP)	75 PD (comparison of in-person vs. VC) 225 PD (comparison of VC vs. smartphone assessments)	Enrolled a large cohort from 42 different states across United States and Canada and allowed for longitudinal comprehensive assessment of PD participants.	Low enrollment to recruitment ratio (sample size of 226 but only 75 completed validation tests).
Omberg et al. (34)	Smartphone application vs. in-clinic testing	Active and passive monitoring of activity and location	PD (1414)	Real-world data and continuous monitoring from smart devices can replace patient recall evaluating treatment efficacy between visits.	Lack of engagement (patterns in mobile research studies tend to mirror use patterns for apps in general)
			Non-PD (8432)		
Weizenbaum et al. (35)	Smartphone	Context, mood, alertness, motivation, Trails-B task and Backwards Spatial Span task assessment on the Mind Learn-Assess-Manage-Prevent app	PD (27) without dementia	The Backwards Spatial Span assessing working memory was predicted by performance on the in-person MoCA, and the smartphone-based WMS-III Spatial Span and Trail-Making Test	Participants had mild to moderate PD, so the results are not generalizable to higher severities.
Chahine et al. (36)	Online self-reported vs. in-person	Non-motor Symptom Questionnaire (NMS-QUEST), Parkinson’s Disease Questionnaire (PDQ)	Online PD (12,654)	Recruit a large database of participants without access to in-person studies	Self-reported characteristics and clarifying questions modify the responses
			In-person PD		
			3 cohorts:		
			(422)		
			(700)		
			(508)		
Bock et al. (37)	Self-reported HRQOL in a large, online cohort	NMS-Quest-PD, Health-Related Quality of Life (HRQOL), MDS-UPDRS Part II	PD (23,058)	A large, diverse online cohort reveals differential effects based on gender, education, and income. Increased participation of PD underrepresented in research.	Analysis was cross-sectional and participants needed to have access to the Internet.

One major method to study cognition in PD online is utilizing VC. For instance, the feasibility and efficiency of using VC methods have been demonstrated using PONT, a Protocol for Online Neuropsychological Testing (27). PONT outlines a thorough protocol from the first steps of recruiting participants via online platforms to conducting online neurological evaluations and testing cognition in PD. PONT enabled researchers to conduct 15 cognitive experiments on PD and control groups, recruiting over 1,500 participants over the course of 3 years. Another recent study utilized the PONT protocol to recruit and test individuals with PD and healthy controls across multiple domains, including cognitive status, emotional well-being, social support, and personality traits (28). The study demonstrated the feasibility and efficiency of utilizing VC and automated online testing to assess multiple nonmotor capacities, producing results comparable to those obtained from F2F testing.

Further testing the applicability of using VC technology in PD research, researchers assessed the feasibility of a VC version of the Montreal Cognitive Assessment (MoCA-VC) administered on PD in comparison to F2F (14). A small cohort of participants (*n* = 11) was evaluated via F2F and then via VC 1 week later. No significant differences were found between the methods, and participants had a median difference of 2 (IQR: 1–2.5) between F2F and VC scores. A study on telerehabilitation of PD compared F2F and online assessment of the dysarthric speech disorder associated with PD, determining that the online method is valid and reliable (30). Another study tested the validity and generalizability of remote administration of the MoCA test (31). The MoCA-VC was administered to English and Hebrew speakers from three different populations: PD, Cerebellar Ataxia, and healthy controls via VC. It was found that the MoCA-VC scores did not differ from traditional F2F studies, demonstrating convergent validity. Second, the MoCA scores of both patient groups were lower than controls, demonstrating construct validity. Third, no performance differences between the two language versions of the MoCA-VC were found, supporting its generalizability to different languages and efficiency in collecting binational data (USA and Israel). In a recent study, PD participants completed the MoCA-VC and an online self-administered math verification task (32), demonstrating an arithmetic deficit in PD patients compared to healthy controls. This study showed that online neuropsychological assessment in PD can have a major contribution to our understanding of the basal ganglia’s (BG) role in cognition.

In addition to VC methods, smartphone-based methods allow real-time measurement of cognition in PD. A recent observational study developed a protocol called Assessing Tele-Health Outcomes in Multi-year Extensions of Parkinson’s Disease Trials (AT-HOME PD) (33). This study established a method for 2 years of virtual assessment of PD participants via smartphone application-based motor assessment, online patient-reported outcomes, and VC-based cognitive assessments. This demonstrates the feasibility of using digital tools such as smartphone applications for longitudinal tracking of PD. Another study utilized the same protocol and compared smartphone-based assessments to in-clinic assessments of PD (34). In 6 months, 960 participants from 50 states performed five self-administered PD symptom assessments, including motor and cognitive assessments (e.g., speeded tapping and memory). Task performance predicted self-reported PD status and correlated with in-clinic Movement Disorder Society-Unified Parkinson’s Disease Rating Scale (MDS-UPDRS) scores.

Additionally, one study evaluated 27 individuals with PD who responded to smartphone notifications five times per day over 10 days (35). The smartphone application assesses two cognitive tasks: the backwards spatial span task and the Trails-B task (35). The smartphone-based backwards spatial span score was predicted by the participant’s performance on the F2F MoCA, and the smartphone-based spatial span and trail making tests, demonstrated convergent validity between traditional F2F tests and smartphone assessment of working memory.

While VC and smartphone methods are efficient, self-reported online surveys have been particularly useful in obtaining large-scale data on PD. One study (36) on PD patients compared a large online self-reported PD sample (*n* = 12,654) to data collected from three observational F2F studies (*n* = 422, 700, 508). Notably, it has been found that PD research typically underrepresents women and minorities, often failing to collect data from diverse, representative samples due to limitations inherent to traditional in-person methods (17, 38). Studies traditionally include patients treated in movement disorder centers, who are predominantly white men of higher socioeconomic status (39). To address this issue, targeted online campaigns to recruit underrepresented minorities have proven to be effective in improving diversity (40). Similarly, a large-scale study evaluated self-reported Health Related Quality of Life (HRQOL) in 23,058 individuals with PD through the Fox Insight Study (37). The authors note that the online recruitment and testing strategies of the Fox Insight Study allowed for the inclusion of patients underrepresented in research, such as people with PD outside of medical centers. These results highlight the potential of online testing to reduce barriers to participation and improve diversity.

Lastly, online forums such as Patients Like Me (41) also allow individuals with PD to engage with their community and self-report their symptoms. Data from such forums, which is more ecological and naturalistic, can advance PD research. One study aggregated the outcomes from this forum reported by patients on different medications to assess treatment efficacy more efficiently than traditional clinical trials, which may be costly and time-consuming (42).

## Advantages and challenges of online cognitive testing in PD

While there are promising developments in online cognitive testing in PD, several challenges exist. Next, we summarize the advantages and challenges of online cognitive testing, discuss important considerations and when possible, offer solutions. We will focus on three major aspects of testing PD patients’ cognitive abilities online: Recruitment and sampling methods, measurement and participation, and disease monitoring and management. For each of the above aspects, we will discuss the main differences between in-person and online testing, considering factors that are relevant both in a research and clinical setting. See Table 3 for a summary of advantages and challenges of online cognitive testing in PD.

**Table 3:** Advantages and challenges of online cognitive testing in PD.

Feature	Advantage	Challenge
Recruitment and sampling methods	Scalability—recruitment methods (e.g., social media) have higher exposure and distribution. Accessible due to reduced time and cost of travel, especially for those with severe motor disability. Faster pace of data collection, allowing larger and more diverse samples. Facilitate multi-center studies and faster data collection in a wide range of geographic areas, allowing better representation of the population. Facilitate sampling from rural areas, especially for rare subtypes of PD, such as genetic subtypes (e.g., LRRK2) or clinical subtypes (e.g., young onset PD). Allowing better representation of underestimated populations, such as those who are not English speakers.	Maintaining and ensuring data privacy—for patients’ information protection, recruitment, and cooperation. Tackling fraudulent participation. Selection bias—recruitment methods (e.g., support groups) are more accessible for patients who have the means and time to access the Internet. Bias toward technology-oriented, literate, and milder disease severity individuals. Individuals with severe impairment may not be able to access the computer/internet and participate in online studies. Low ratio between sample size/enrollment: A large sample is interested in participating, but a low percentage of the recruitment pool completes the studies (e.g., 20–40%). This is lower than the 50% attrition rate in F2F tests (43).
Measurement and participation	Allow to incorporate modification in testing to the specific needs of PD patients. Sessions can be shorter and more spread out, which can be less demanding for patients with strict medication regimens or severe motor impairments. Hypothetically, there would be less stress on patients when testing at home/without supervision vs. when tested in clinic/laboratory settings. This needs to be tested. Instant results and automatic scoring.	Different cognitive impairments and fatigue evident in PD can impair the performance in online measures, potentially leading to underestimations of participant abilities. It is more difficult to clarify task instructions, especially for individuals with language or general cognitive deficits, which is not rare in PD. Variability in the testing environment and devices (e.g., computers), which can increase noise in the data. The potential caregiver’s involvement could influence test results and impact the quality of the data. Online platforms and internet connectivity issues can cause delays and interruptions that may skew testing results. Currently, validation data is largely missing. Currently, psychometrics data is largely missing.
Disease monitoring and management	Time savings for both the client and clinician. Allows pre-appointment cognitive testing. Allows post-appointment cognitive testing. Longitudinal follow-up and studies allow better disease characterization as PD is a neurodegenerative condition and more testing sessions can be conducted online. Allows comprehensive assessments across various cognitive domains on the same participants. Tools can be utilized to develop early screening for PD faster. Allows to compare between populations within a single study, enhancing conclusions about tools’ sensitivity and specificity. Allow clinicians more time and cost-effective monitoring and disease management.	It is challenging to verify the identity and diagnosis of the participant. Unclear if online motor severity assessment is adequately sensitive and specific. Unclear if online cognitive tests measure the same constructs as in-person tests – there may be a conceptual difference in the construct tested in online studies. Currently, normative data is largely missing.

### Recruitment and sampling methods

Scalability is a critical consideration, especially when recruiting and testing participants with neurological conditions such as PD, which affects 1% percent of the population above age 60 (44). Online methods allow efficient recruitment necessary for large-scale studies (45, 46). Methods for improving scalability in recruitment include using social media platforms (e.g., Facebook) and support groups (27), which may be leveraged for extensive exposure and distribution (31). Online advertisements, such as sponsored ads, can also attract a diverse pool of participants.

While online recruitment can increase studies’ sample size and be time and cost-effective, ensuring privacy remains a challenge. Maintaining privacy is a significant concern, especially for individuals with PD, as studies often require extensive medical history (47). Participants often mention privacy issues as their main concern when providing feedback for online studies (14, 33, 36). In-person studies at a specific research laboratory or clinic may establish more trust than studies utilizing VC or smartphone-based methods. Participants may be wary of fraudulent online activity and thus feel less inclined to disclose personal medical information through an online platform.

Thus, we also outline recommendations for maintaining data privacy in online patient testing. While not specific to PD, multiple resources devoted to digital privacy in online research can be found in the literature (48–50). In general, recommendations are region-specific. We focus on the unique legal frameworks in the United States, Europe, and Israel. In the USA, the Health Insurance Portability and Accountability Act (HIPAA) establishes a fundamental layer of protection for identifiable health information. This act is crucial in online patient testing, providing guidelines for securing sensitive patient data. In Europe, the General Data Protection Regulation (GDPR) serves as the primary legislation for personal data protection, with far-reaching implications for data security in online patient testing. In Israel, the Privacy Protection Law, 5741-1981, acts as enforcement for personal digital information, regulating data collection and storage practices in online patient testing. Note that these recommendations provide a starting point for region-specific regulations governing health data. However, they should not be considered exhaustive, and further consultation with legal experts in each jurisdiction is advised to ensure full compliance.

We also provide concrete recommendations for different experimental stages. First, one technique to minimize privacy risks is random participant ID assignment. Each participant should be assigned a unique identifier instead of their real name during data collection. This allows researchers to track individual data while maintaining anonymity from the initial stages of data collection. Second, we propose limiting data collection to measures relevant to cognitive tasks only, minimizing the risk associated with the storage of excess personal information. Third, restricting access to patient data is critical, and access should be granted solely to a limited number of directly involved researchers. Fourth, de-identification of the data is necessary before analysis, including the removal of personal identifiers, such as emails. Fifth, secure platforms for online data collection and storage are crucial for ensuring privacy. These platforms typically employ encryption measures to further safeguard participant information.

Along with privacy concerns, it is also important to consider the possibility of deceptive incentive-driven participation in online PD studies. Therefore, conducting a thorough medical history, including information about medical providers who have diagnosed the patient, the institution where they receive medical care, and detailed symptom history, is critical. For instance, in a recently developed protocol for online neuropsychological testing (PONT), we proposed concrete steps to conduct comprehensive cognitive and motor evaluation specifically to PD (27).

Accessibility is a key advantage of online studies, reducing travel time and expenses. Commuting can be burdensome, particularly for those with PD who have motor and mobility limitations (8). VC, smartphone, or self-report online cognitive surveys could reduce this accessibility bias. One important advantage is the increased capacity for sampling from rural areas. PD F2F research frequently comprises participants from the same geographic area, often metropolitan cities (15, 51, 52). Thus, studies on PD can benefit from recruitment of rural populations (53). In contrast, online testing can greatly increase geographical diversity, streamlining multi-center studies and data collection across nations (28, 45, 46). This allows for the inclusion of rare subtypes of PD, such as genetic variants or early-onset PD, as well as inclusion of populations rarely reached from developing countries.

However, it is important to note that reliable internet access in rural areas is a constraint of online testing. Broadband internet access in rural areas of the United States becomes less available the more rural the region (54). The high cost of internet infrastructure and maintenance, particularly in remote regions such as Sub-Saharan Africa, may present a barrier to online testing (55). Thus, while online cognitive testing may improve overall accessibility within relatively developed areas, internet access and technology are not always available in more underdeveloped parts of the world.

Online cognitive testing’s accessibility benefits have global implications. Online testing facilitates the collection of data across different languages and cultures, providing an opportunity to efficiently expand research participation across geographic and language barriers. International studies using in-person testing methods require extensive collaboration and bureaucracy in order to collect PD patient data from multiple testing sites, while online testing can be conducted from the comfort of the participant’s and researcher’s home (31). In addition, by including more than one language, especially non-English speakers, researchers can better capture the cognitive abilities of speakers of different languages and more accurately characterize cognitive dysfunction on a global scale (56).

Online testing of cognitive abilities has several challenges in terms of recruitment and sampling. A major challenge with online testing is variability from technical resources, which could create more noise in the online data. This noisy data may lead to a reduced representation of the cognitive abilities of the population (57). This is especially true for PD participants, given their motor impairments, such as tremors, which can increase unexplained variability when measuring cognition. Also, while online testing has the potential to reach a wider group of participants and increase representation, we recognize the limitation of variation in access to technology, which may hinder some participants with little to no access to the internet.

Another main challenge for online recruitment and sampling is selection bias. Patients participating in support groups or social media may have better internet access, technology literacy, access to technological resources, or more time than others. Researchers can mitigate this bias by using diverse recruitment strategies and transparently reporting their sampling method. There may also be a bias against those with severe cognitive or motor impairments, limiting their ability to use their computers. In these instances, researchers and clinicians can consider hybrid alternatives, such as phone interviews [e.g., using the telephone-adapted MoCA (58)].

Another notable challenge is the low completion rate. Online studies often have a low ratio between the final sample and the initially enrolled participant pool (59). Researchers may initially recruit a large PD sample, but only a low percentage of this recruitment pool completes each study (e.g., 20%–40%), as individuals may drop out or be excluded for a variety of reasons, including lack of interest in ongoing research participation, privacy concerns, or unfamiliarity with the required technology (10, 27, 33). Smartphone-based studies are known to be hindered by high dropout rates, especially for studies that require more than one session (60). For example, in AT-HOME PD, a longitudinal virtual observational study of PD participants, 226 participants initially enrolled in the study, but only 75 participants (33%) completed all assessments (33). To increase the engagement of PD participants, the STEADY PD III Recruitment Committee suggests raising awareness through targeted campaigns. For this study, the researchers developed a “Recruitment Toolkit” which included the following patient engagement materials: (1) Appointment cards; (2) Patient Education Frequently Asked Questions (FAQs); (3) Site Brochures; (4) Site Posters; (5) Patient geared Slide Deck; (6) Patient Talking Points; (7) Site Press Release; and (8) Thank You Cards (61).

### Measurement and participation

In addition to recruitment, the measurement of cognitive abilities and participation may be improved via online methods. When adapting F2F cognitive testing tools to an online format, careful task modification is essential. This presents an opportunity to incorporate modifications tailored to the specific needs of patients, such as those with PD. For example, recent studies adjusted specific items in the MoCA for the online format (27), taking into consideration the motor limitations associated with PD (8). Online tasks can prioritize the measurement of accuracy and require fewer button presses to accommodate motor limitations, as opposed to focusing on measuring reaction time, which is more prone to motor-related variability.

In addition to motor limitations, PD patients frequently have non-motor symptoms, such as cognitive impairment, visual hallucinations, and fatigue. Cognitive impairments are common in PD patients and include impairments in specific domains such as working memory, task switching, and decision-making (11). Additionally, visual hallucinations could be exacerbated by screen time, further complicating online test validity (62). Fatigue can decrease motivation and engagement, impacting test completion and reliability (9, 63). To reduce the potential effect of these three non-motor factors, we propose that tasks should take a maximum of 30 min to complete and include breaks every 10 or 15 min. Notably, online testing provides the flexibility to create short-duration tasks and segmented assessments across several sessions.

Another common challenge in online testing is how to clarify task instructions when needed. The cognitive or language deficits associated with PD can make comprehension a challenge, especially when delivered remotely (64, 65). Studies have identified various language impairments in PD, including word-finding difficulties and struggles with complex sentences (66, 67). Such deficits can hinder online test performance, particularly in tasks that require reading comprehension or verbal fluency to interpret complex task instructions. These challenges underscore the importance of presenting clear and simple instructions to ensure comprehension among those with PD. Taking into consideration these comprehension deficits in PD, we propose that task’s instructions and visual stimuli should be simple, clear, and in a large font size to better suit these older patient populations.

An additional consideration is the testing environment. An unfamiliar lab setting may introduce bias (68), since participants tested F2F may experience more stress than those who participate online from the comfort of their homes. The laboratory environment may exacerbate motor symptoms, particularly in PD, which is known to exhibit worsened symptoms in stressful conditions (69, 70). However, considering the effect of online testing environments is also important, as it can introduce unexplained variability and impact results (13, 71). Home environment could be especially challenging for PD participants with attention deficits or cognitive impairments (72). To address this issue, online attention checks can be added to the test. A hybrid approach may be useful, where some steps occur via VC and conversational agents, such as chatbots or avatars, used to add a conversational continuous aspect to the interaction. These approaches have already been implemented in the clinical setting through apps such as ONParkinson, which provides personalized resources to patients with PD and their caregivers via a chatbot (73).

Lastly, accounting for variability arising from technical differences in online testing is crucial. For instance, internet connectivity and speed can impact time-sensitive measures, such as response time. Moreover, differences in hardware components, including screen size, resolution, and audio output, can introduce noise into the data (74).

### Disease monitoring and management

Online cognitive testing can also have major implications for disease monitoring and management of PD patients. For neuropsychological assessment in progressive conditions such as PD, long-term, consistent monitoring of symptoms is necessary (29). Longitudinal online assessments can allow early detection of cognitive decline, track alterations in symptoms, assess medication efficacy, and address decline (or improvement) in patient quality of life over time.

Online testing protocols could be useful in characterizing multiple cognitive and psychological capacities (28). Since PD has a multifactorial effect on nonmotor domains, ranging from emotional well-being to cognition (75–79), online studies can measure multiple domains, allowing for better characterization of PD and the disease progression over time. Online cognitive testing also facilitates between-group comparison (28, 31), which can improve sensitivity and specificity of assessment tools. However, although online protocols may be adapted for several domains of study, it may not be suitable for all domains. For example, olfaction, especially since olfactory dysfunction has been previously noted in PD patients (80).

A major challenge with characterization of PD online is accurately assessing the degree of disability (1, 51). Therefore, developing a standardized protocol that adapts traditional cognitive (and motor) in-person assessments to online settings is crucial. For instance, multiple online VC protocols have been developed to assess cognitive symptoms in PD using the MoCA (14, 29, 31).

Additionally, verifying the identity and diagnosis of patients can pose another challenge in disease characterization. There is a tradeoff between relying on human-computer interaction, which increases efficiency and reduces bias, vs. human-human interaction, which could feel more authentic and meaningful to participants and allows for greater flexibility and sensitivity. Human-human interaction may be more useful for engaging PD participants, particularly regarding a reluctancy to disclose their condition-specific details. One solution for this problem is an initial assessment via a VC platform (e.g., Zoom) to establish a personal connection and assess symptoms. During this meeting, researchers and clinicians can obtain information about disease onset, progression, treatment regimens, and participants can use this opportunity to ask any questions they may have (27).

However, compared to in-person, online interpersonal interactions have several limitations, including: (1) Reduced nonverbal cues, such as body language; (2) Reduced emotional connection: In-person interactions allow for shared physical space, touch, and proximity; (3) Technological Barriers such as technical glitches, delays, and audio/video quality issues can disrupt the flow of conversation; (4) Screen-Mediated Interaction can creates a psychological barrier that makes online interactions feel more transactional than relational; (5) Privacy Concerns: Participants may feel less comfortable discussing personal matters online since in-person interactions offer a sense of confidentiality and safety.

Notably, accurate disease characterization typically requires experts such as neuropsychologists and neurologists, but those are limited, especially for people who live in remote areas. Thus, due to limited healthcare resources and the increasing prevalence of neurodegenerative conditions (81), accessing consistent care can pose a significant challenge. Online testing could serve as a bridge by providing a time and cost-effective tool to monitor disease progression and facilitate disease management (46, 53).

## Conclusion

Currently, online cognitive testing in people with PD poses many challenges. These challenges include the absence of validation and normative data, the generation of noisy cognitive data due to motor impairments, fraudulent participation, limited interpersonal interactions, and uncertainty regarding whether online cognitive tests measure the same constructs as in-person tests. Nevertheless, the emergence of online cognitive testing has expanded the horizons of PD research. It could facilitate multinational and multilingual studies and enable comparisons across multiple cognitive domains and patient groups. By collecting large, diverse, and longitudinal datasets, we can more effectively map the progression of cognitive symptoms in PD.

We hope that as technology advances, PD testing will achieve a balance between efficiency and genuine human connection in online cognitive testing. Recent studies underscore technology’s potential to revolutionize not only PD research but also clinical diagnoses, ultimately benefiting people with PD in their day-to-day challenges. Online assessment could diminish geographical barriers, enhance healthcare accessibility, and have significant implications for remote patient monitoring and disease management worldwide.

## Author contributions

SB: Conceptualization, Investigation, Methodology, Writing – original draft, Writing – review & editing. ALA: Writing – original draft, Writing – review & editing. YBA: Visualization, Writing – original draft, Writing – review & editing. LM-K: Conceptualization, Project administration, Supervision, Writing – original draft, Writing – review & editing. WS: Conceptualization, Investigation, Methodology, Project administration, Resources, Supervision, Validation, Visualization, Writing – original draft, Writing – review & editing.
